# Disruption of androgen receptor-cofactor interactions by the RNA-binding protein FUS/TLS alters androgen signalling in prostate cancer

**DOI:** 10.1038/s41388-026-03682-3

**Published:** 2026-02-06

**Authors:** G. N. Brooke, D. A. Leach, R. L. Culley, A. Azadova, L. Latonen, E. Rees, M. A. Alkheilewi, A. C. Pine, F. M. Fioretti, C. S. Reader, S. M. Powell, V. Reebye, J. Waxman, T. Visakorpi, C. L. Bevan

**Affiliations:** 1 School of Life Sciences, University of Essex, Wivenhoe Park, Colchester, ESSEX UK; 2 Department of Surgery and Cancer, Imperial College London, London, UK; 3 Institute of Biomedicine, University of Eastern Finland, Kuopio, Finland; 4 Faculty of Medicine and Health Technology, Tampere University and Tays Cancer Center, Tampere University Hospital, Tampere, Finland; 5 Fimlab Laboratories, Tampere, Finland

**Keywords:** Mechanisms of disease, Cancer

## Abstract

Prostate cancer is dependent upon the androgen receptor (AR), the activity of which is modified by cofactors that either enhance or repress its activity, often in a context-dependent manner. FUS/TLS is a multifunctional protein known to be important in multiple cancer types; in prostate cancer, we previously showed that FUS has a potential tumour suppressor role. Here, transcriptomic analysis of the LNCaP prostate cancer cell line shows a significant overlap in genes regulated by FUS and the androgen receptor. We demonstrate that FUS can regulate androgen receptor activity, in either direction, but predominantly represses androgen signalling. Reporter assays and domain-specific analyses of FUS identified mechanisms by which FUS modifies androgen receptor activity. FUS interacts with the androgen receptor and other cofactors to repress transcription; ChIP assays suggest that repression occurs via disassembly of the transcriptional complex. Quantitative proteomics and RNA-Seq were used to investigate FUS expression in patient samples across prostate cancer stages. FUS was found to be down-regulated in primary tumours, but up-regulated in advanced aggressive stages. These findings suggest that in early prostate cancer, FUS represses AR activity and tumour progression, leading to its down-regulation. In contrast, increased FUS expression in advanced disease appears to be linked to a loss of AR regulatory control.

## Introduction

Prostate cancer (PCa) is the second leading cause of cancer-related death in Western men and tumour growth is almost invariably dependent upon the androgen receptor (AR), a ligand-dependent transcription factor and member of the steroid receptor subgroup of the nuclear receptor superfamily [1]. In common with other nuclear receptors, the AR has a modular structure consisting of N- and C-terminal activation functions (AF-1 and AF-2, respectively), a central DNA-binding domain and a C-terminal ligand binding domain. In the absence of androgen, the AR exists in the cytoplasm as part of a large heat shock protein complex that holds the receptor in a ligand-binding competent state. Upon ligand binding the receptor undergoes a conformational change that promotes nuclear localisation, dimerisation and binding to response elements within the regulatory regions of target genes, resulting in gene expression changes which in the case of PCa can drive tumour growth [1]. These transcriptional effects involve the recruitment of cofactor proteins to liganded, DNA-bound AR.

Therapies for non-organ confined PCa target the androgen signalling axis by either blocking androgen synthesis (e.g., LHRH antagonists/analogues and CYP17A antagonists) or directly targeting the receptor (antiandrogens) [2]. Antiandrogens inhibit AR activity via multiple mechanisms, for example by targeting the receptor to specific cellular compartments and/or by promoting the recruitment of corepressors rather than coactivators [3]. These therapies are successful in the majority of patients but invariably eventually fail and the tumours progress to the castrate resistant (CRPC) stage. Much evidence exists to suggest that the AR continues to drive CRPC growth, even in the androgen-depleted environment [4]. In addition to constitutive or promiscuous activation of AR itself, it has been proposed that alterations in cofactor levels, over-expression of coactivators or down-regulation of corepressors, can enhance AR signalling and promote therapy failure [5–7]. Interestingly, many of these cofactor proteins are also regulated in response to androgen signalling. Prohibitin, for example, is a co-repressor of AR activity and is down-regulated in response to androgen, while some co-activators are androgen-upregulated at the mRNA level [8–10]. The AR therefore regulates multiple factors that subsequently feedback and regulate its own activity.

In a proteomic screen to identify AR regulated proteins, we identified that the RNA-binding protein Fused in Ewings Sarcoma/Translocated in Liposarcoma (FUS/TLS) is down-regulated in response to androgen and appears to have the characteristics of a novel tumour suppressor in PCa [11]. Immunohistochemical analysis of primary prostate tumours demonstrated that FUS expression inversely correlated with Gleason grade and directly correlated with survival. Further, manipulation of FUS expression was demonstrated to have a significant bearing on PCa proliferation in in vitro and in vivo models, with FUS having potent inhibitory effects. Additional studies have found FUS to have similar activity in other cancer types. For example, Chen et al. [12] found FUS to have tumour suppressor activity in pancreatic cancer. In contrast, FUS levels have been found to be increased in liposarcoma, and elevated levels of this RNA-binding protein correlate with poor prognosis in non-small cell lung cancer (NCLSC) [13, 14]. FUS therefore appears to play alternative roles in different malignancies.

FUS is a multifunctional protein, shown to be important in multiple cellular processes including DNA repair, RNA splicing, microRNA processing and transcriptional regulation [15]. In the latter process, FUS has been demonstrated to regulate gene transcription via non-coding RNA-dependent recruitment to the promoter regions of target genes. For example, Wang et al. found that FUS is recruited and tethered to the *Cyclin D1* promoter via non-coding RNA, disrupts the recruitment of accessory proteins such as p300 and subsequently blocks gene transcription [16]. FUS has also been previously demonstrated to interact with the DBD of other steroid receptors, but this interaction does not affect receptor-DNA binding [17]. Further, FUS is also recruited to the DNA-bound oestrogen receptor (ER)-α when the receptor is bound to the antagonist tamoxifen [18] suggesting that it may act as a corepressor of ERα.

FUS has previously been shown to interact with the AR and to enhance receptor activity in some contexts [17, 19]. Here we show that FUS can enhance AR regulation of some genes, but predominantly inhibits AR activity through disruption of the transcriptional complex. The inhibitory effects of FUS upon PCa growth [11] are therefore likely to be, at least in part, via direct repression of AR activity. Interestingly, FUS levels are elevated in CRPC, and this is appears to be linked to a loss of AR regulatory activity.

## Methods

### Ligands

Ligands were dissolved in EtOH (VWR, Leicestershire, UK) and stored at −20 °C. 17β-oestradiol, dexamethasone, progesterone, bicalutamide and enzalutamide were from Sigma (Dorset, UK), mibolerone (MIB) from PerkinElmer (Buckinghamshire, UK). HDAC inhibitors Trichostatin A (dissolved in EtOH), valproic acid and sodium butyrate (both dissolved in ddH_2_O) were from Sigma.

### Plasmids

The following have been previously described: pSG5-SRC1e, ERE-LUC, pSG5-ERα [20]; PB-PROM-LUC and TAT-GRE-E1B-LUC [21]; pSVAR [22]; pSG5-ARA70, pCMVβ-p300 [23]; pCMV-HA-EWS [24]; pSG5-GR [25]; pGL2-LEXA-GAL4-LUC (luciferase reporter under control of both LexA and Gal4 response elements), pSG5-LEXA-VP16 (LexA DNA-binding domain fused to VP16 activation domain) [26], p5-GAL-LUC, pVP16-AR [27] and pSG5-hPR-B [28]. Full-length and truncated FUS were PCR-cloned into the pM-GAL4 (Gal4 DNA-binding domain) (Clontech, Mountain View, CA, USA) plasmid using 5’ *Bam*H1 and 3’ *Xba*1 restriction sites. TAF15 was PCR cloned from pTL-TAF15 into pSG5 using 5’ *Bam* HI and 3’ *Xma* I restriction sites (primer sequences provided in Supplementary Tables 1 and 2). All plasmids were verified by sequencing. pTL-TAF15 and pCMV-HA-EWS were kind gifts from Prof L Tora (Institut de Génétique et de Biologie Moléculaire et Cellulaire, France) and Prof. P Cohen (University of Dundee, Scotland) respectively. The FUS mutants (G92A, F438V, G488S and K510E) were generated using the QuikChange II Site-directed mutagenesis kit (Agilent, Santa Clara, CA, USA). Mutagenesis primer sequences are provided in Supplementary Table 3. All plasmids were verified by sequencing.

### Cell culture

Cell lines were obtained from American Type Culture Collection (ATCC, Manassas, VA, USA), except for PC3wtAR, which was a kind gift from Prof A. Cato [29]. Lines were STR authenticated by the ATCC and were frozen at a low passage. Lines were grown for a maximum of 6 months before a fresh aliquot was defrosted. Mycoplasma testing was performed every 3 months using the MycoAlert Detection Kit (Lonza, Basel Switzerland). COS-1 and MCF-7 cells were cultured in Dulbecco’s modified Eagle’s medium. LNCaP, C42, C42B, PNT1A, BPH1, DU145, PC3, PC3wtAR and 22RV1 cells were cultured in RPMI 1640 as described previously [27, 30]. LNCaP-FUS and LNCaP-TR2 were cultured in RPMI containing tetracycline-free FCS and Penicillin-Streptomycin-Glutamine (PSG) [11]. For experiments requiring hormonal stimulation, cells were incubated in phenol-red free media containing double charcoal stripped FCS (First Link UK, Birmingham, UK) and PSG prior to treatment. For proliferation assays, 22RV1 were seeded in hormone-depleted medium, transfected with pEGFP-Empty or pEGFP-FUS, treated ± MIB and proliferation of GFP-positive cells monitored over time using an Incucyte Cell Imaging System.

### Reporter assays

COS-1, PC3wtAR and MCF-7 cells were seeded at 60% confluence in 24-well plates and incubated for 24 h in androgen-depleted media. For dose–response experiments, COS-1 cells were transfected using the modified calcium phosphate method [27] with 100 ng of expression vectors for the AR, GR, PR, and ERα, 0–400 ng of FUS, EWS or TAF15 expression vectors, 1 μg of luciferase reporter (TAT-GRE-E1B-LUC, PB-PROM-LUC or ERE-LUC) and 100 ng of PDM-LACZ-β-GAL PC3wtAR and MCF-7 cells were transfected using FuGENE 6 (Promega, Madison, WI, USA) with 100 ng PDM-LAC-Z-β-GAL and 1 μg TAT-GRE-E1B-LUC and 0–400 ng of pSG5-FUS.

For the trans-activation/repression and mammalian 2-hybrid assays cells were transfected with 1 μg of luciferase reporter (pGL2-LEXA-GAL4-LUC or 5-GAL-LUC respectively) and 200 ng of pM-GAL4-FUS (FULL-E). For the mammalian 2-hybrid assays cells were co-transfected with 100 ng pVP16-AR, pSG5-SRC1e, pCMVβ-p300 or pSG5-ARA70. For the trans-repression assays cells were co-transfected with 100 ng pSG5-LEXA-VP16. In all transcription assays cells were co-transfected with 100 ng of PDM-LACZ-β-GAL, and up to 400 ng of pSG5-FUS or pM-GAL4-FUS (FULL-E) per well.

The amount of DNA added to each well was made equal by the addition of pSG5-empty or pM-GAL4-EMPTY. After transfection cells were incubated ± ligand/HDAC inhibitor for 24 h before lysis, and luciferase and β-galactosidase activities measured using the LucLite Plus (PerkinElmer) and Galacto-light Plus (Tropix, Bedford, MA, USA) kits, respectively.

### Microarray and qPCR

LNCaP-FUS and LNCaP-TR2 were cultured in hormone-depleted media for 72 h ± 10 nM Doxycycline and treated ±10 nM Mibolerone for a further 24 h. Cells were lysed with QIAshredders, and RNA recovered using an RNAeasy kit with the additional DNAse step (Qiagen, Hilden, Germany). RNA quality was checked using a BioAnalyser (Agilent, Santa Clara, CA, USA) and cDNA synthesised via reverse transcription using a Super Script II kit (Invitrogen, Carlsbad, CA, USA). Microarray was performed by Source Bioscience (Nottingham, UK) using Human HT-12 v4 Beadchips (Illumina, Saffron Walden, UK). Data was analyzed using AltAnalysis software [31]. Gene regulation was set as *p* < 0.05, and a log fold change greater than 0.5 or below −0.5. Heatmaps were generated using Morpheus software (Broad Institute) with hierarchical clustering performed using Pearson correlation coefficients. Gene Ontology analysis was performed using DAVID [32].

For qPCR, RNA was extracted and cDNA generated as described and performed using an ABI 7900HT qPCR machine (Applied Biosystems, Foster City, CA, USA). qPCR was performed using Fast SYBR Green (Applied Biosystems) and primers specific to *KLK3*, *NDRG1*, *FUS*, *DMC1*, *TMPRSS2* and *L19* (primer sequences provided in Supplemental Table 4). Relative gene expression changes were determined using the 2^-∆∆CT^ method and gene expression normalised to *L19* data.

Analysis of H3K27Ac in LNCaP cells was accomplished using previously published data (GSE51621 [33]) and pipelines [34]. H3K27Ac sites were determined using the Snakemake analysis pipeline via cistrome, BWA and SAM tools for read mapping/filtering/sorting, and peaks were called with MACS2 (*q* < 0.01) [35]. A cutoff of fivefold signal-background ratios was used to filter peaks. The peak data was combined with the above microarray data to perform BETA analysis, creating regulatory potential scores for each gene in relation to binding peaks [36].

### Immunoblotting and immunofluorescent imaging

Immunoblotting was performed as previously described [5] using the following primary antibodies: AR (N-20), FUS (4H11), EWS (G-5) antibodies were from Santa Cruz (CA, USA), SRC-1 antibody (21952-1-AP) was from Proteintech (IL, USA). The TAF15 antibody (GTX77901) was from GeneTex (Irvine, CA, USA) and the acetylated H3 antibody (06-599) was from Merck Millipore (MA, USA).

For imaging, COS1 and LNCaP cells were seeded in hormone-depleted media on glass coverslips in 24 well plates. COS1 cells were transfected with 200 ng of pSVAR and pSG5-FUS per well using FuGENE 6. Cells were incubated for 24 h (COS1) or 72 h (LNCaP) and treated ±10 nM Mibolerone for 2 h. Cells were fixed with 2% paraformaldehyde for 15 min, washed 3 x with PBS (5 min with rotation) and permeabilised using 0.1% Triton X-100 (10 min). Cells were again washed 3 x with PBS prior to blocking with 1% BSA in PBS-0.05% Tween for 30 min. Cells were incubated with primary antibodies specific for AR and FUS followed by HRP or ALEXA Fluor conjugated secondary antibodies and visualised as previously described [37].

### Immunoprecipitation

COS1 cells were seeded in full media and transfected with pEGFP-AR + pSG5-FUS/pSG5 Empty. Cells were left for 48 h and then GFP-AR purified using GFP-TRAP magnetic beads, following the manufacturer’s instructions (ProteinTech). AR and FUS levels were visualised using immunoblotting.

### GST pull-down

AR domains (AF-1, DBD, AF-2, AF-2H) were expressed in BL21-codon plus *Escherichia coli*. Successful protein induction was confirmed using SDS-PAGE and Coomassie blue staining. The GST-fused constructs were purified and incubated with PC3wtAR lysate as previously described [30], and FUS interactions investigated using immunoblotting.

### Chromatin immunoprecipitation

LNCaP-FUS cells were grown to approximately 70% and serum starved for 72 h. Cells were treated ±10 nM Mibolerone for 2 h before cross-linking with formaldehyde (Sigma) for 10 min at 37 °C. ChIP was performed as previously described [38]. The AR (N-20), SRC-1 (M-341) and FUS (4H11) antibodies were from Santa Cruz and the RNA Pol II (CTD4H8) antibody was from Merck Millipore. qPCR was performed to investigate AR, SRC1, RNA Pol II and FUS recruitment to the *PSA* enhancer (forward 5′-TGA CAG TAA ACA AAT CTG TTG TAA GAG ACA-3′; reverse, 5′-AGC AGG CAT CCT TGC AAG AT-3′).

### Depletion of FUS levels using siRNA

FUS levels were reduced in LNCaP cells using a Dharmacon On-Target siRNA pool (L-009497-00-0005; Thermo Fisher Scientific, MA, USA) as previously described [27].

### Immunohistochemistry

Formalin-fixed, paraffin-embedded tumour microarrays of PCa (*n* = 239) and CRPC (*n* = 100) samples were used. The clinicopathological information of the cohort is described in Leinonen et al. Sections were deparaffinized and antigen retrieval was performed by using sodium-citrate buffer (pH 6) at +98 °C for 15 min. The staining was performed by Lab Vision Autostainer (ThermoFischer Scientific Inc., Waltham, MA, USA). FUS (4H11) was used as the primary antibody followed by secondary antibody (N-Histofine® Simple Stain MAX PO; Nichirei, Tokyo, Japan). ImmPACT DAB (Vector Laboratories, Burlingame, CA, USA) was used as a chromogen. The sections were counterstained with hematoxylin and mounted with DPX mounting medium (Sigma-Aldrich). Scoring of staining intensity on tumour areas was performed on a 0–3 scale, and the difference in score distributions between PCa and CRPC groups was statistically assessed with Chi squared test.

### RNA-Seq and Proteomic analysis of patient samples

Tissue specimens from 10 BPH, 17 untreated PC, and 11 CRPC samples were acquired from Tampere University Hospital (Tampere, Finland). Five 5 µm slices of fresh-frozen tissue were processed as previously described and digested peptides analysed using a Nano-RPLC-MSTOF instrumentation using Eksigent 425 NanoLC coupled to high speed TripleTOF™ 5600 + mass spectrometer (Ab Sciex, Concord, Canada) [39]. For transcriptomic analysis, RNA was extracted from matching samples and libraries prepared for paired-end analysis (Illumina HiSeq2000) [40]. Alterations in the expression of FUS, at different stages of PCa, was subsequently assessed in the proteomics and RNA-seq datasets.

## Results

### The androgen receptor and FUS commonly regulate a number of genes

**Fig. 1:**
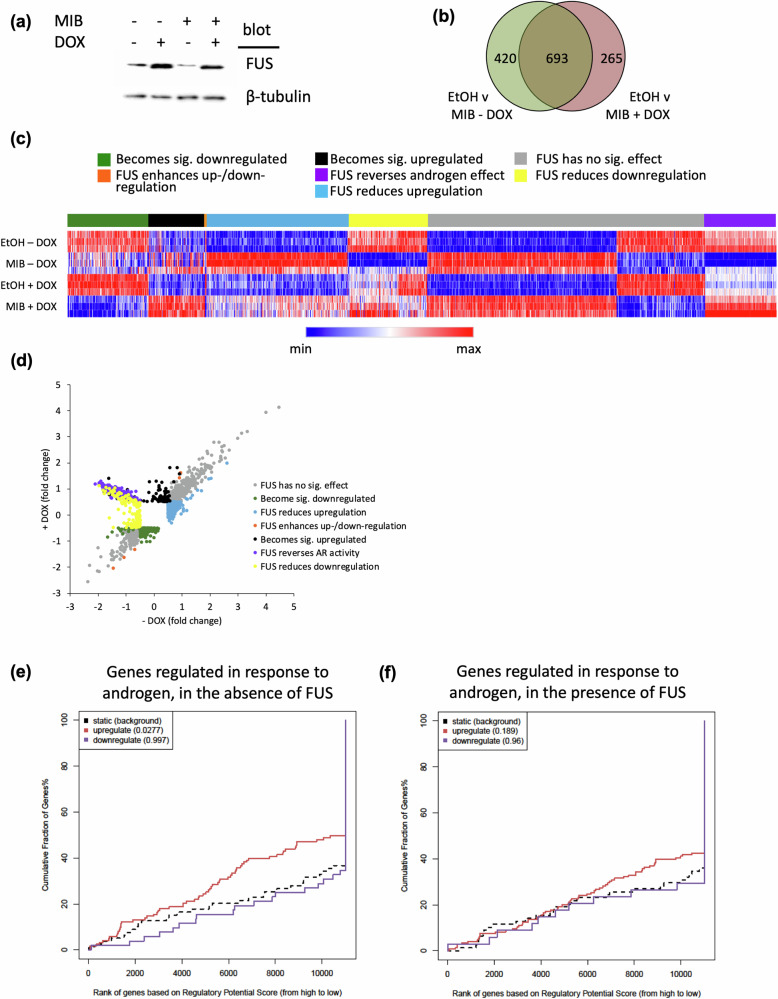
Cross-talk between the androgen receptor and FUS. **a** LNCaP-FUS cells were treated ± mibolerone (MIB) ± doxycycline (DOX) for 24 h and enhanced FUS expression confirmed using immunoblotting. Microarray analysis was performed on LNCaP-FUS to identify genes differentially expressed in response to MIB alone or plus FUS ( + DOX). **b** Venn diagram summarising the number of genes regulated by FUS and/or MIB. **c** Heat map visualisation of the 1378 genes found to be differentially regulated in response to MIB and/or DOX. Each horizontal line represents one sample. **d** Scatter plot indicating the alterations in androgen-regulated gene expression in response to elevated FUS ( + DOX). Genes up-regulated in response to androgen and regulated by FUS were cross-referenced with H3K27Ac ChIP-Seq data from LNCaPs (GSE51621). Correlation between genes up-regulated by AR in the (**e**) absence or (**f**) presence of FUS.

We have previously demonstrated that FUS is a potent inhibitor of androgen-dependent tumour growth in PCa models and that its expression is directly correlated with patient survival [11]. To identify down-stream targets of FUS, transcriptomic analysis was performed on the LNCaP-FUS cell line; LNCaP cells stably transfected with a doxycycline inducible *FUS* expression vector resulting in a robust inducible increase in FUS protein levels (Fig. 1a). Cells were hormone starved for 3 days and then treated ± mibolerone (synthetic analogue of dihydrotestosterone) ± doxycycline for 24 h. Mibolerone was used at a final concentration of 10 nM, which we have found to induce maximal activity [11]. As a control, the parental line (LNCaP-TR2) was also treated with the same conditions. Treatment of LNCaP-TR2 with doxycycline significantly altered expression of only 1 androgen-regulated gene (LOC147804, Supplemental Fig. 1). This was removed from the subsequent analysis of the LNCaP-FUS line to ensure that any changes identified in response to doxycycline are as a result of FUS over-expression and not an artefact of doxycycline treatment.

In the absence of doxycycline, expression levels of 1113 genes were regulated (644 up, 469 down) in response to androgen (*p* < 0.05, FC>log2(0.5), Fig. 1b–d). Gene ontology analysis found that these androgen-regulated genes (ARGs) are representative of a range of pathways, with many being involved in metabolism (Supplemental Fig. 2). FUS reduced androgen-induced up-/down-regulation of 429 genes. The expression of 265 genes, which in the absence of elevated FUS showed weak androgen regulation (*p* > 0.05 and/or FC < log2(0.5)), became significantly androgen-regulated (108 up- and 157 down-regulated) following over-expression of FUS, while the expression levels of 5 target genes already significantly androgen-regulated was enhanced by FUS (Fig. 1c). Most interestingly, the direction of androgen regulation of 140 genes was reversed in response to FUS expression, i.e., genes that were androgen down-regulated were found to be androgen up-regulated following doxycycline treatment. Gene ontology analysis of AR target genes regulated by FUS found pathways such as “steroid hormone biosynthesis” and “protein processing in endoplasmic reticulum” to be altered (Supplementary Fig. 2). The expression of 420 of the androgen-regulated genes was not significantly altered by FUS.

To see if histone modifications might explain the differential regulation of target genes by FUS, the gene expression data was compared to acetylated H3K27Ac ChIP-seq data (representing active regulatory regions) from LNCaP treated with androgen (GSE51621) [33]. We focussed on ARGs that were found to be altered in response to FUS over-expression. In the absence of FUS, genes up-regulated by androgen were, as expected, found to significantly correlate with H3K27Ac (Fig. 1e). However, when FUS was over-expressed, this association was lost (Fig. 1f). This was specific for genes that were up-regulated in response to androgen, with no association observed for genes that are down-regulated in response to AR signalling. Where genes were only responsive to androgens in the presence of FUS, there was also no association with H3K27Ac (data not shown).

**Fig. 2:**
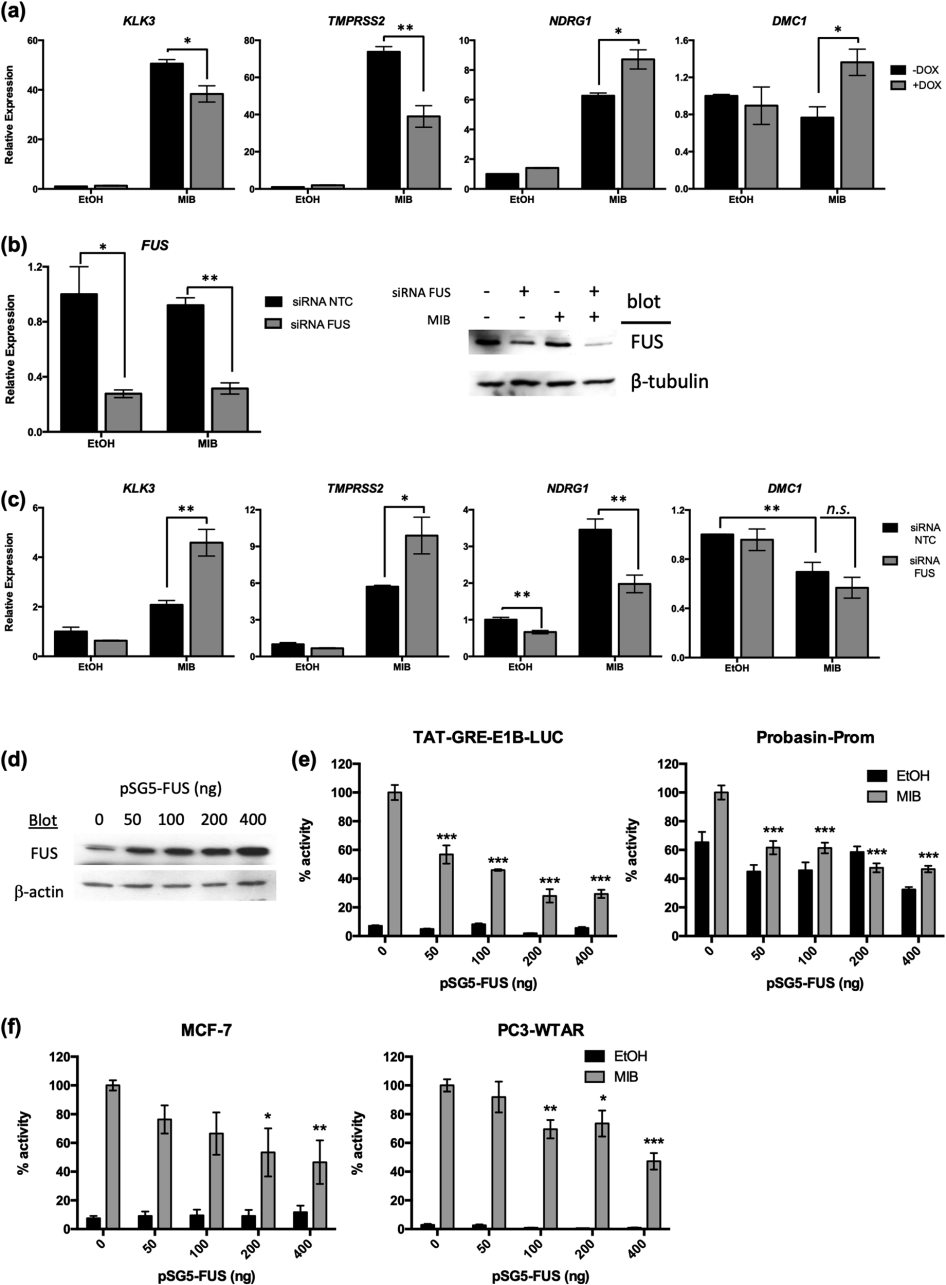
FUS regulates androgen receptor activity. **a** LNCaP-FUS cells were treated ± mibolerone (MIB) ± doxycycline (DOX) for 24 h and qPCR performed to quantify gene expression. **b** LNCaP cells were transfected with siRNA targeting FUS and successful depletion confirmed using qPCR and immunoblotting. **c** Target gene expression, following siRNA depletion of FUS was measured using qPCR. **d, e** COS-1 cells were transfected with plasmids encoding the AR, androgen-responsive luciferase reporters (TAT-GRE-E1B-LUC or PROBASIN-PROM-LUC), a β-galactosidase expression vector, increasing amounts of pSG5-FUS and treated ± MIB (mibolerone). **d** Immunoblotting was performed to confirm FUS over-expression and **e** luciferase assays were performed to investigate the effect of elevated FUS levels upon AR activity. **f** MCF-7 and PC3wtAR were transfected with the TAT-GRE-E1B-LUC reporter, β-galactosidase expression vector, increasing amounts of pSG5-FUS and treated ± MIB. Luciferase activity was normalised to β-galactosidase activity and expressed as a percentage of AR activity in the presence of MIB and absence of FUS. Mean ± 1SE of 3 independent repeats performed in duplicate. ANOVA *p < 0.05, **p < 0.005.

To validate the transcriptomic data, a panel of targets, from the different categories of regulated genes, was validated using qPCR following FUS over-expression (Fig. 2a) or FUS depletion (Fig. 2b and c). Two known AR-upregulated target genes, *KLK3* and *TMPRSS2* [41, 42], were found to be repressed by FUS; as expected these were up-regulated when FUS was depleted. Likewise, expression of an AR-target gene, *NDRG1*, whose androgen-regulation was enhanced in response to FUS overexpression, was reduced following FUS knock-down (Fig. 2a and c). The expression of *DMC1*, a gene down-regulated in response to androgen, was found to be significantly enhanced in response to mibolerone + doxycycline, also in agreement with the microarray data (FUS reverses androgen effect, Fig. 1c (purple)). In summary, the data demonstrates that FUS has differential effects upon AR signalling, but appears to predominantly repress AR activity.

### FUS is a novel repressor of androgen receptor activity

The findings from our previous study [11] and microarray data presented here suggest that FUS inhibits PCa growth, at least in part, via regulation of androgen signalling. To further investigate FUS regulation of AR activity, COS-1 cells were co-transfected with plasmids encoding the AR, one of two different androgen responsive luciferase reporters and increasing amounts of FUS expression plasmid, which produced increasing amounts of FUS protein (Fig. 2d). AR activity was reduced dose-dependently, by approximately 70% and 50%, in response to FUS over-expression on the TAT-GRE-E1B-LUC and PROBASIN-PROM-LUC promoters respectively (Fig. 2e). To confirm that this effect was not cell-line dependent or an artefact of exogenous transiently expressed AR, reporter assays (TAT-GRE-E1B-LUC) were repeated in AR-positive MCF-7 and PC3wtAR (PC3 cells stably transfected with wild-type AR [29]) cells. In both cell types, FUS again significantly repressed AR activity (Fig. 2f).

**Fig. 3:**
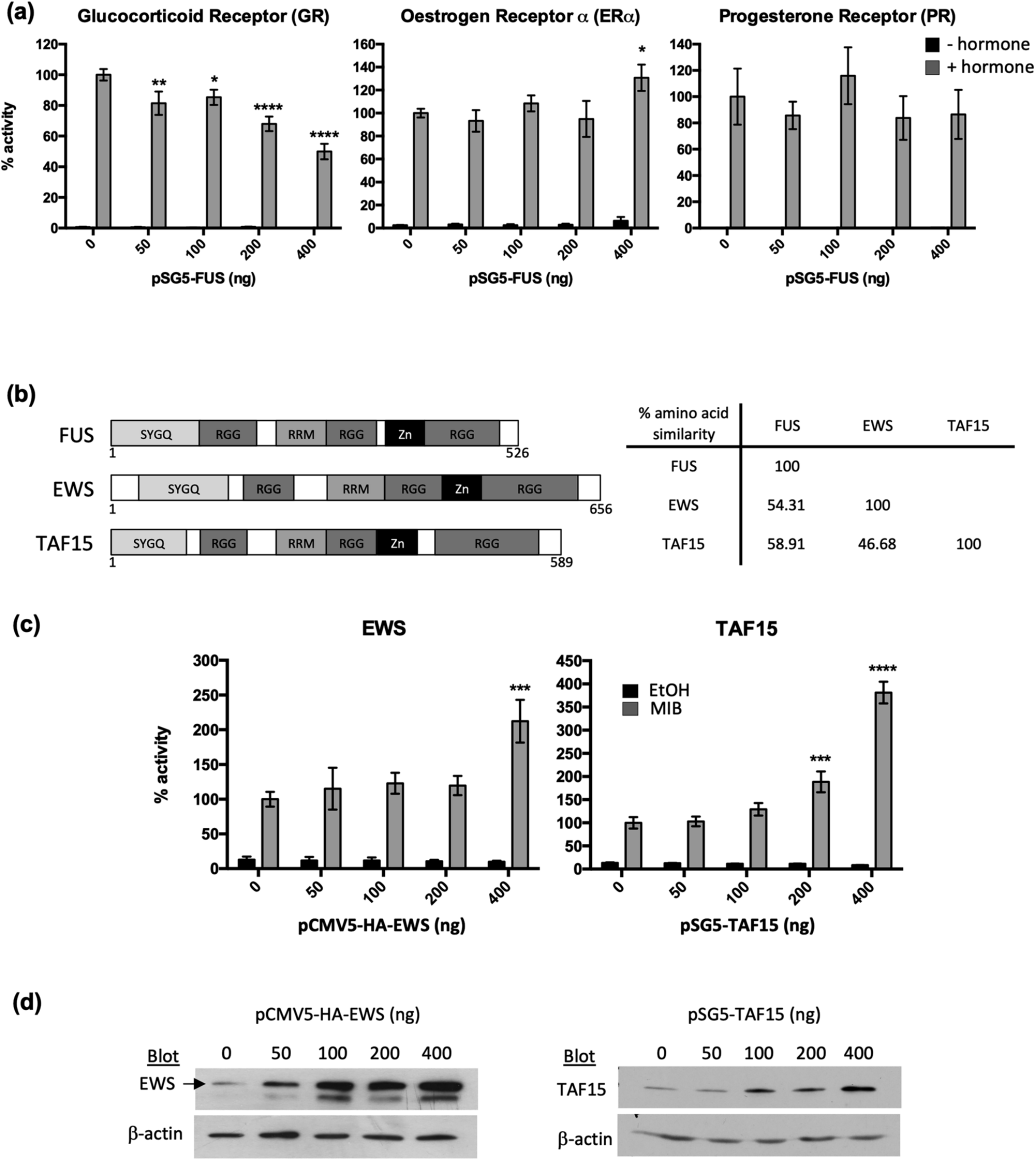
FUS represses the glucocorticoid receptor, whereas EWS and TAF15 enhance AR activity. **a** COS-1 cells were transfected with an appropriate luciferase reporter plasmid (GR, PR, AR = TAT-GRE-EIB-LUC and ERα = 3xERE-LUC), a β-galactosidase expression vector (BOS-β-gal) and expression plasmids for the GR, ERα, PR and FUS. Cells were treated with the receptors cognate hormone (GR = dexamethasone, ERα = oestradiol, PR = progesterone) for 24 h. Luciferase data was normalised to β-galactosidase and expressed as a % of receptor activity in the absence of FUS. **b** Schematic representation of the TET family members highlighting the key domains and table summarising their % amino acid similarity: SYGQ - serine-tyrosine-glycine-glutamine domain; RRM - RNA-recognition motif; Zn - zinc finger domain; RGG - Arginine-Glycine-Glycine- rich domains. **c** Luciferase assays were performed as above with pSVAR and increasing amounts of vectors coding for EWS and TAF15. **d** Cells transfected with increasing concentrations of plasmids encoding EWS and TAF15 were lysed, proteins separated by SDS-PAGE and protein expression visualised using immunoblotting. Results are mean ± 1SE of 3 independent experiments repeated in duplicate. ANOVA *p < 0.05, **p < 0.005.

As described previously, FUS interacts with several steroid receptors, but the effect upon the transcriptional activity of the receptors was not assessed [17]. To determine whether the repressive effects of FUS are specific for AR, the effect of increasing FUS levels upon the transcriptional activity of the closely related glucocorticoid, oestrogen and progesterone receptors was investigated. Similar to the AR, the activity of glucocorticoid receptor was repressed, whereas little effect upon progesterone and oestrogen receptor-α ’s activity was evident; only a slight increase in oestrogen receptor-α activity was measured in response to high FUS levels (Fig. 3a).

FUS, along with TAF-15 and EWS, is a member of the TET family of RNA-binding proteins. TET family members have similar domain structures, including an RNA recognition motif (RRM) and zinc finger (Zn), sharing amino acid sequence homology of 47–59% (Fig. 3b). Reporter assays were therefore performed to investigate whether the other TET family members also regulate AR activity. Interestingly, in contrast to FUS, EWS and TAF-15 were found to enhance AR transcriptional activity (Fig. 3c, d).

### FUS interacts with the AR via its RNA Recognition Motif domain

**Fig. 4:**
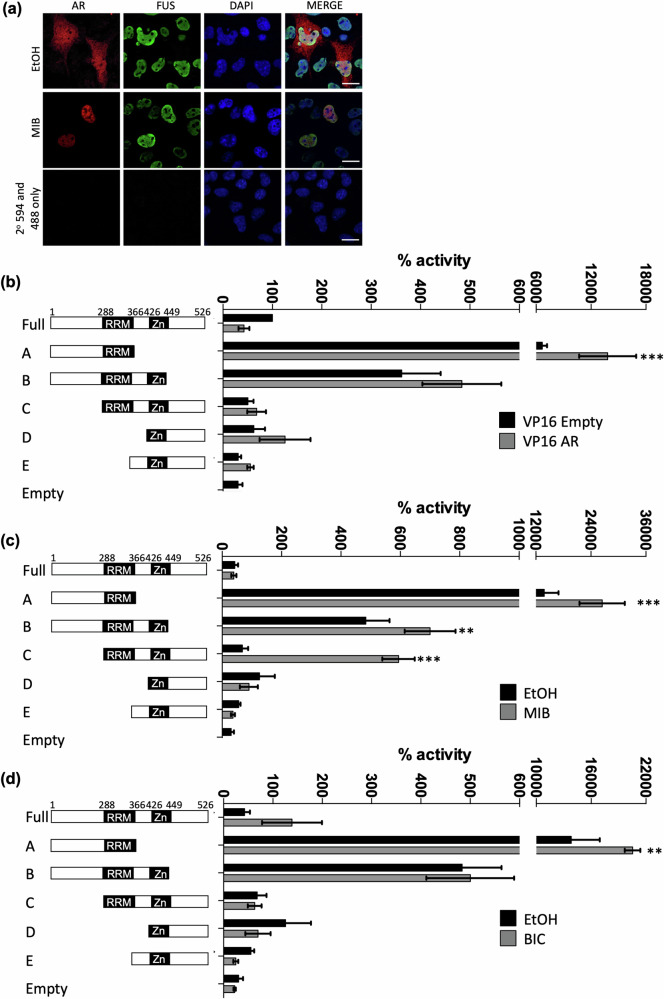
The AR co-localises and interacts with FUS. **a** COS-1 cells were seeded in hormone-depleted media for 24 h and transfected with expression vectors for the AR and FUS or empty control. Cells were treated for 2 h ± mibolerone (MIB). Nuclei were stained with DAPI and cellular localisation visualised using fluorescent microscopy (bars = 20 µM). **b** COS-1 cells were transfected with plasmids encoding a Gal-4 responsive luciferase reporter (5-GAL-LUC), full-length or truncations of FUS fused to Gal-4 ± VP-16 fused AR, **c** ± MIB, **d** ± BIC (bicalutamide). Luciferase data was normalised to β-galactosidase activity. Mean of 3 independent repeats performed in duplicate ± 1SE. ANOVA *p < 0.05, ** *p* < 0.005, *** *p* < 0.0005, comparing black to grey bars for each construct.

To characterise the mechanism(s) of regulation of AR by FUS, we first investigated its cellular localisation. In COS1 cells transfected with expression vectors for AR and FUS, AR protein was cytoplasmic and nuclear in the absence of androgen, and translocated into the nucleus upon hormone stimulation, as expected (Fig. 4a). The FUS antibody detected the endogenous and exogenous protein, showing it to be localised in the nucleus and unaffected by androgen treatment. Similar results were observed when the experiment was repeated using endogenously expressed proteins in LNCaP (Supplemental Fig. 3).

As some degree of colocalization in the nucleus was evident, mammalian 2-hybrid assays were performed to investigate if FUS and AR interact. The experiment utilised full-length and truncated forms of FUS fused to Gal4 DNA-binding domain (Gal4DBD) and VP16 activation domain (VP16AD)-fused AR (Fig. 4b). As previously described [19, 43, 44], the N-terminus of FUS (construct A, amino acids 1-366) contains a potent activation domain, which resulted in auto-activation of the co-transfected Gal4-responsive reporter. C-terminal extension (construct B, amino acids 1-449) reduced this intrinsic activity. Comparison of VP16-empty and VP16-AR identified ligand-independent interaction between the AR and FUS construct A (amino acids 1-366), but not other constructs. To see if FUS interacts with the AR when agonist-bound, the experiment was repeated in the presence of mibolerone (Fig. 4c). The interaction between the AR and construct A was enhanced in the presence of mibolerone, and additional ligand-dependent interactions were identified between the AR and the other RRM-containing constructs B and C (amino acids 1-449 and 288-526 respectively). The interaction between FUS and the AR therefore appears to be dependent upon the RRM (amino acids 288-366).

**Fig. 5:**
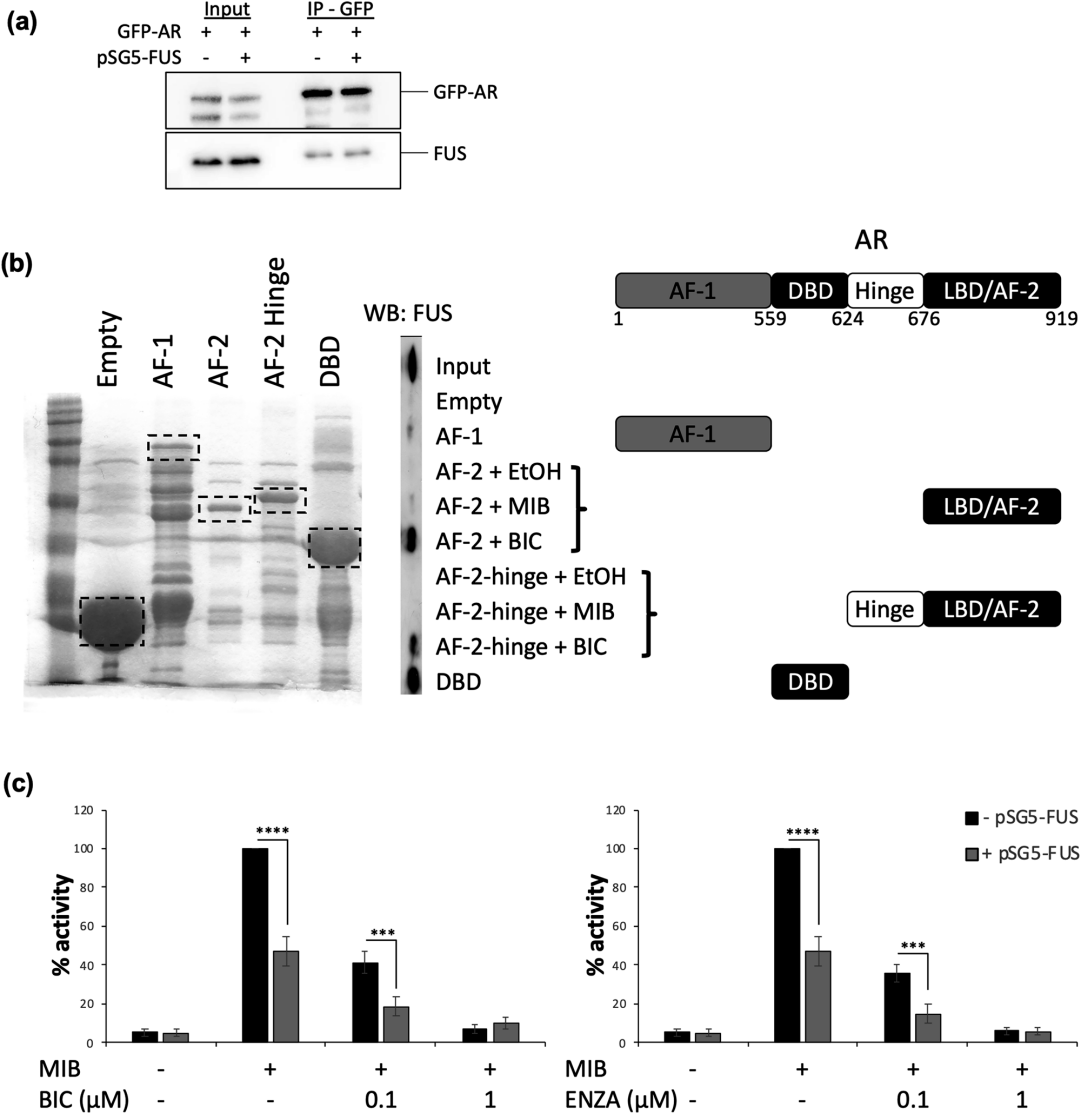
FUS interacts with the AR and enhances the antagonistic activity of antiandrogens. **a** COS-1 cells were transfected with plasmids encoding GFP-tagged-AR and FUS. GFP-tagged AR was immunoprecipitated using GFP-trap beads and successful AR pull-down verified by immunoblotting. **b** GST-tagged domains of AR were expressed in bacteria. Successful expression was confirmed by SDS-PAGE and Coomasie staining, The constructs were incubated with PC3 lysate and immunoblotting performed to confirm successful FUS pull-down. **c** COS-1 cells were transfected with plasmids encoding the AR, FUS, a luciferase reporter (TAT-GRE-E1B-LUC) and β-galactosidase and treated ± MIB ± BIC or ± ENZA (enzalutamide). Luciferase data was normalised to β-galactosidase activity. Mean of 3 independent repeats performed in duplicate ± 1SE. ANOVA *p < 0.05, ** *p* < 0.005, *** *p* < 0.0005, comparing black to grey bars for each construct.

Antagonists have been shown to inhibit AR activity, at least in part, via recruitment of co-repressors [45]. To investigate if FUS interacts with antiandrogen-bound AR, mammalian 2-hybrid assays were repeated in the presence of the antiandrogen bicalutamide. Similar to the interaction induced by mibolerone, the antagonist-bound AR was found to interact with FUS construct A, however unlike mibolerone, no interactions were evident for constructs B and C (Fig. 4d). To validate the interaction, a GFP pull-down immunoprecipitation was carried out with GFP–AR as the bait and FUS as the co-immunoprecipitated prey. In agreement with the mammalian 2-hybrid assays, FUS was found to interact with the AR (Fig. 5a). To investigate which regions of the AR interact with FUS, GST pull-downs were performed using different domains of the AR (Fig. 5b). Interestingly, the AF-2/AF-2 hinge region was found to interact with the FUS in the presence of the antiandrogen bicalutamide, as did the DBD in a ligand independent manner. Next, reporter assays were performed in the presence of bicalutamide or enzalutamide (BIC/ENZA, Fig. 5c). As expected, FUS and BIC/ENZA each inhibited AR activity. Importantly, the combination of FUS and BIC/ENZA showed additive effects, resulting in over 80% repression of AR activity compared to less than 40% individually.

### FUS contains activation and repression domains

**Fig. 6:**
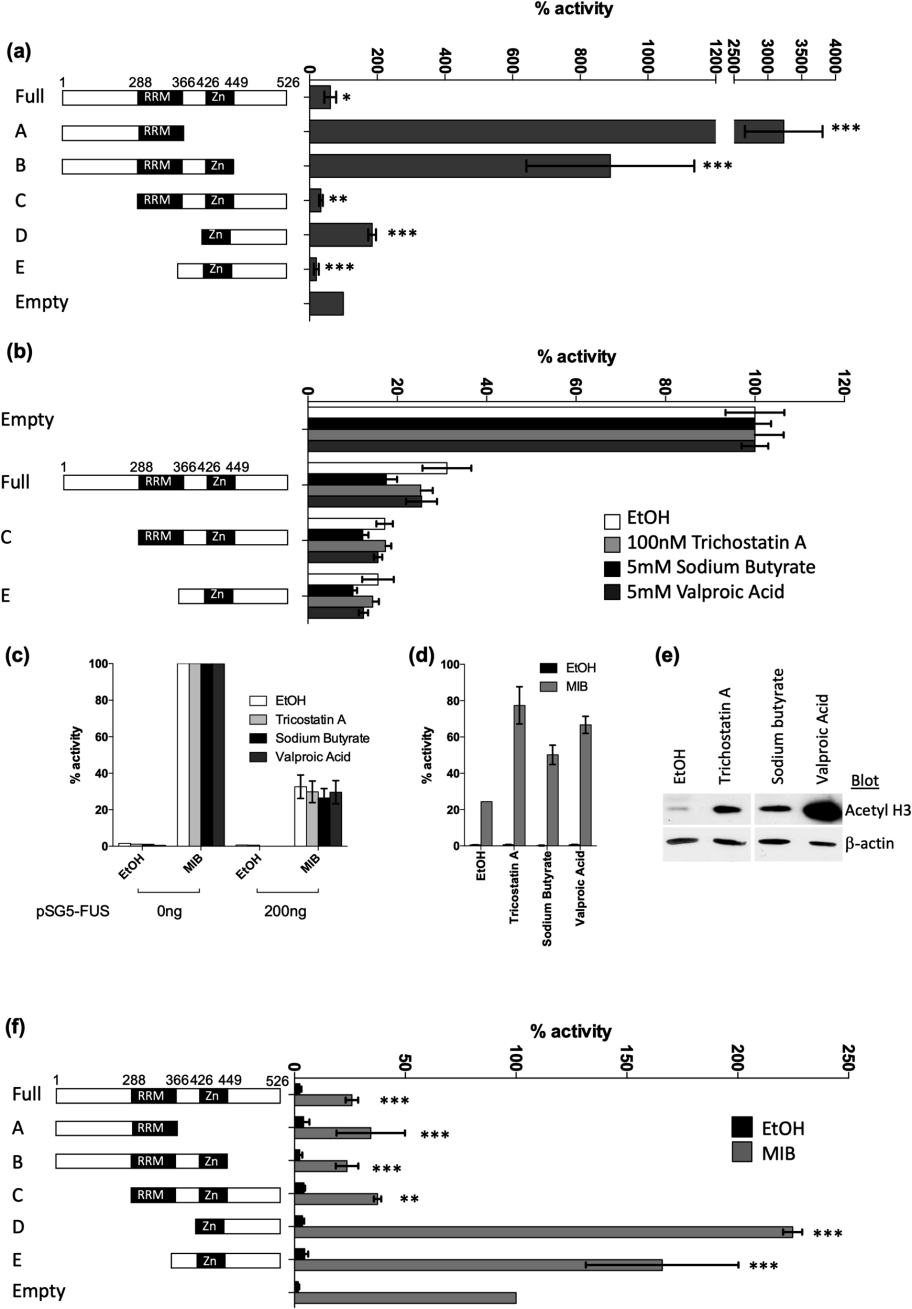
FUS represses AR activity via an HDAC-independent mechanism. **a** COS-1 cells were co-transfected with a luciferase reporter construct containing 7xLexA and 4xGal4 response elements (LexA_(7)_-Gal4_(4)_-LUC) and expression plasmids for β-galactosidase, LexA-VP16, and either Gal4-DBD alone (Empty) or Gal4-DBD fused to full-length or domains of FUS. **b** The trans-repression assay was repeated for full-length FUS and constructs C and E and cells were treated with the HDAC inhibitors trichostatin A, sodium butyrate, and valproic acid. Values are expressed as a percentage of VP16-LexA activity in the presence of empty Gal4-DBD and are the mean + 1SE of 3 independent experiments performed in duplicate. **c**, **d** COS-1 cells were transfected with an AR-responsive luciferase plasmid, and expression vectors for β-galactosidase, AR and FUS. Luciferase activity was expressed as % of AR activity in the absence of HDAC inhibitor and FUS. **e** COS-1 cells were treated with HDAC inhibitors and histone H3 acetylation levels visualised using immunoblotting. **f** COS-1 were transfected with AR-responsive luciferase plasmid, and expression vectors for β-galactosidase, AR and either Gal4-fused full-length or truncations of FUS. Luciferase activity expressed as % AR activity in the presence of Gal4-empty plasmid. Mean ± 1SE of 3 independent repeats in duplicate. ANOVA ** *p* < 0.005, *** *p* < 0.0005.

We have shown that FUS can repress AR activity but it also contains intrinsic activation domains. To see if FUS contains intrinsic repression domains, the ability of the Gal4DBD-fused FUS constructs to repress VP16AD-induced luciferase activity were assessed in trans-repression assays [28]. Again, constructs A, B and D auto-activated the system indicating the presence of activation domains (Fig. 6a). Conversely, full-length FUS and constructs C and E significantly reduced VP16-induced luciferase expression (43, 66 and 80% reduction in activity compared to empty vector respectively) indicating overall repressive function. Comparison of constructs C, D and E, also comparing B to A, suggests that the region between the RNA recognition motif and zinc finger (amino acids 366-426) contains a repression domain. Upon removal of this domain (construct D compared to E), repressive effects were abolished.

Several corepressors reduce receptor activity by recruiting histone deacetylases (HDACs) (e.g., [46, 47]). To test this, the trans-repression assay above was repeated with full-length FUS and those constructs demonstrated to have repressive activity (C and E), in the presence of the HDAC inhibitors trichostatin A, sodium butyrate and valproic acid (Fig. 6b). These compounds did not reverse the inhibitory action of FUS constructs, neither did they have any effect on the extent of repression by full-length FUS in COS cells co-transfected with this and wild-type AR (Fig. 6c). As a control, AR activity (Fig. 6d) and histone H3 acetylation (Fig. 6e) were enhanced in response to the HDAC inhibitors demonstrating that they are functional in this system. Therefore the mechanism by which FUS represses AR activity does not appear to be via recruitment of Class I/II HDACs.

To identify which domains were necessary for AR repression, the Gal4-fused FUS constructs were co-transfected with full-length AR and an androgen-responsive luciferase reporter (TAT-GRE-EIB-1-LUC) (Fig. 6f). Full-length FUS and constructs A-C repressed AR activity by over 60% whereas the C-terminus of FUS (constructs D and E) was found to enhance AR activity. Interestingly the intrinsic repression domain (amino acids 366-426) was not required for the repression of AR since construct A, which lacks this region, was still able to repress AR activity. Instead, the critical FUS domain for AR repression is the RRM, the region identified to be critical for the FUS-AR interaction (Fig. 4b and c).

### FUS interacts with AR coactivators and disrupts complex formation at response elements

**Fig. 7:**
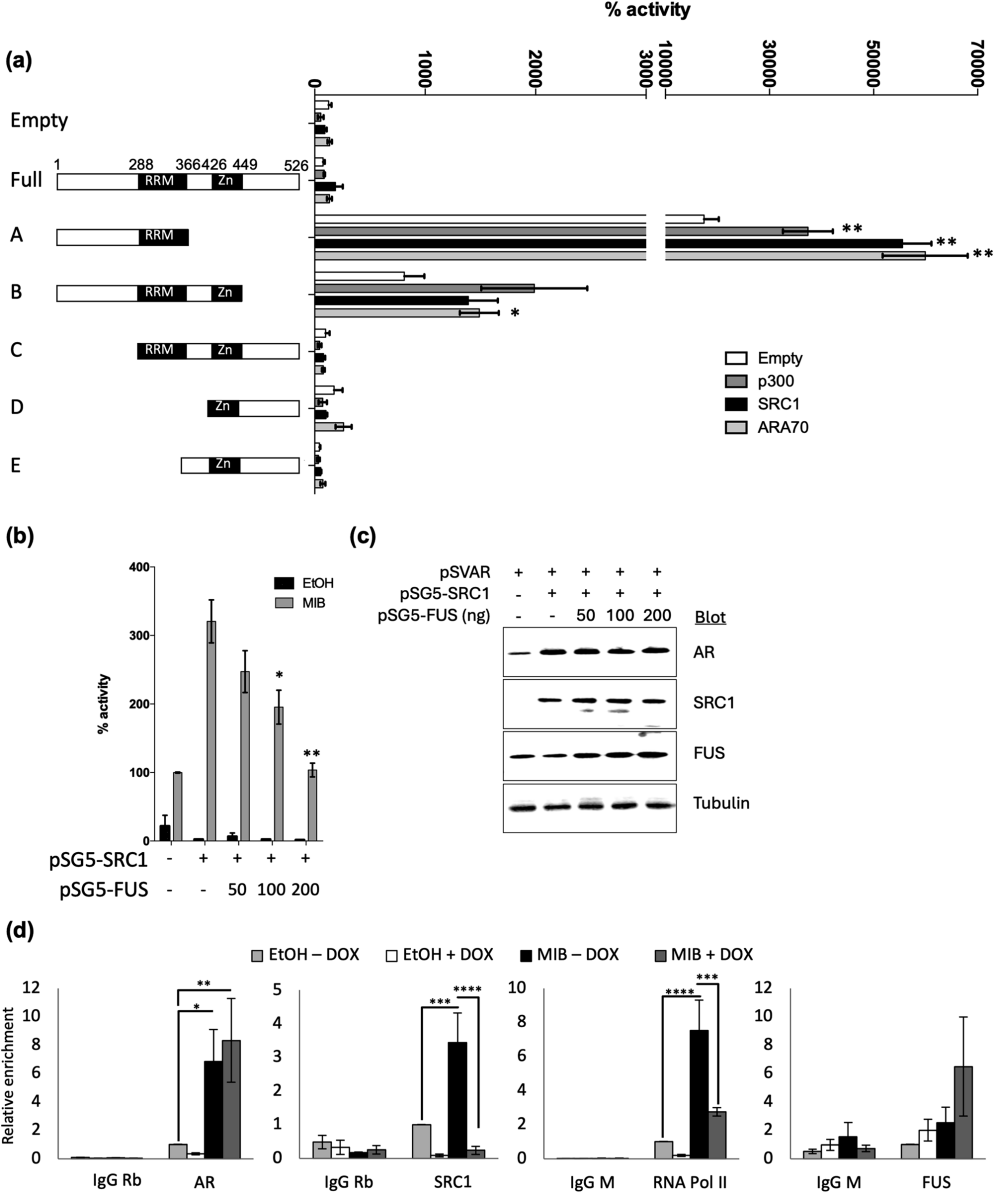
FUS interacts with coactivators and blocks AR recruitment of SRC1 and PolII. **a** COS-1 were transfected with a Gal4 luciferase reporter and β-galactosidase expression plasmid, expression plasmids for Gal4 fused full-length of truncations of FUS and p300, SRC1 or ARA70. **b** COS-1 were transfected with plasmids encoding the AR, SRC-1, FUS and β-galactosidase, and an AR-responsive luciferase reporter. Luciferase activity was expressed as a % of AR activity in the presence of mibolerone (MIB) and absence of SRC1 and FUS. Mean ± 1SE of 3 independent repeats in duplicate. **c** Immunoblotting was performed to confirm successful expression of the AR, SRC1 and FUS. **d** LNCaP FUS were treated ± MIB ± DOX and chromatin immunoprecipitation performed using antibodies specific to the AR, SRC1, RNA Pol II, FUS, or IgG control. Enrichment of the *KLK3* enhancer was analysed using qPCR. Mean ± 1SE of 3 independent repeats. ANOVA * *p* < 0.05, ** *p* < 0.005, *** *p* < 0.0005.

The N-terminus of FUS has previously been demonstrated to interact with and inhibit activity of CBP and p300 [16], cofactors that enhance AR activity [48]. To test FUS interaction with coactivators, a modified mammalian 2-hybrid assay was performed whereby the ability of the coactivators to enhance Gal4DBD-FUS activity was used as an indicator of a positive interaction. The coactivators p300, SRC1 and ARA70 were all found to interact with the FUS N-terminus (construct A, Fig. 7a). It is therefore possible that AR and FUS compete for coactivators, either through direct interaction or sequestration. To investigate this, reporter assays were performed in COS-1 cells transfected with plasmids encoding androgen-responsive luciferase reporter, AR, SRC1 and increasing concentrations of pSG5-FUS. SRC1 enhanced AR activity over 3-fold and increasing concentrations of FUS significantly decreased this enhanced activity (Fig. 7b). AR appeared to be stabilised in the presence of SRC1; AR and SRC1 levels were not altered in response to increasing FUS expression (Fig. 7c). FUS can therefore compete with or sequester co-activators to decrease AR activity.

FUS was previously demonstrated to bind to specific regions of the genome via non-coding RNA [16] and we have demonstrated that FUS interacts with AR and coactivators. Chromatin immunoprecipitation was therefore performed to investigate if FUS affects the assembly of the transcriptional complex. Upon stimulation with androgen, AR, RNA POLII and SRC1 recruitment was enhanced to the regulatory region of *KLK3* (Fig. 7d, MIB vs EtOH, both -DOX). Interestingly, increased FUS expression ( + DOX) did not affect AR recruitment, but did significantly reduce SRC1 and RNA Pol II recruitment. No significant difference was evident for FUS itself binding chromatin, although there was a trend towards increased FUS binding following FUS over-expression. We therefore conclude that FUS represses AR activity not by disrupting AR chromatin-binding, but via disruption of the formation of an active transcriptional complex at the regulatory regions of target genes.

### FUS levels are down-regulated in PCa, but elevated in CRPC

**Fig. 8:**
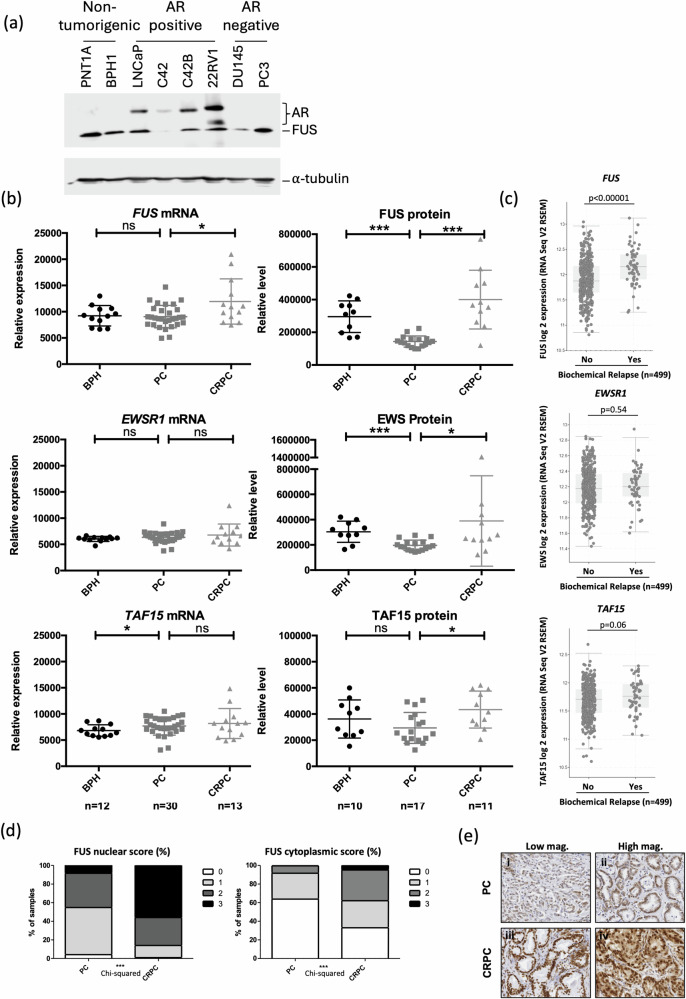
FUS expression is decreased in PCa but increased in CRPC. **a** Immunoblotting was performed to assess AR and FUS expression across a panel of prostate cancer cell lines and non-tumourigenic controls. **b** FUS, EWS and TAF15 expression was analysed in patient samples from BPH, PCa and CRPC at the RNA level (using RNA-seq) and protein level (quantitative mass spectrometry). Mann-Whitney test, * *p* < 0.05, *** *p* < 0.0005. **c** FUS, EWS and TAF15 expression was correlated with biochemical relapse in the TCGA dataset (*n* = 499). **d** IHC was performed on samples from PCa and CRPC using a FUS-specific antibody (Santa Cruz, 4H11). Samples were scored for cellular localisation and intensity. Chi-Squared Test *** *p* < 0.0001. **e** Representative IHC images showing images of weak FUS staining in PCa (i and ii) and high nuclear staining or nuclear and cytoplasmic staining in CRPC (iii and iv).

Alterations in cofactor expression has been proposed as a mechanism of therapy resistance in PCa [1]. We therefore investigated FUS expression across a panel of prostate cell lines representing different stages of the disease (Fig. 8a). FUS was found to be lower in all of the cancer cell lines compared to the non-tumorigenic control PNT1A, with the exception of PC3. FUS levels were lowest in the LNCaP derivative C42, and DU145 cells. To expand on this analysis, the expression levels of FUS, and the other members of the TLS family (EWS1 and TAF15), were analysed at the RNA (RNA-sequencing) and protein (mass spectrometry) levels in clinical samples representing Benign Prostatic Hyperplasia (non-malignant control) (BPH), PCa (untreated, primary tumour) and CRPC (advanced, therapy-resistant) (Fig. 8b).

In agreement with our previous findings [11], FUS protein levels were lower in primary PCa compared to BPH. However, this was not the case at the RNA level. Further, contrary to what was expected, FUS was significantly elevated at the RNA and protein level in CRPC compared to untreated primary PCa (Fig. 8b). As described previously, EWS and TAF15 enhanced AR activity in reporter assays (Fig. 3c). Alterations in EWS and TAF15 levels during disease progression were less pronounced compared to FUS. However, the expression of all family members was found to be significantly higher at the protein level in CRPC compared to PCa (Fig. 8b). To further investigate the role of the TLS family in PCa, in particular therapy resistance, the TCGA dataset was interrogated. Increased FUS expression was found to significantly correlate with biochemical relapse, but no such correlation was found for EWS and TAF15 (Fig. 8c).

To validate the changes in FUS expression in PCa, IHC was performed on samples from 239 patients with primary PCa and 100 with CRPC; slides were scored for nuclear and cytoplasmic intensity. In agreement with RNA-seq and mass spec data, nuclear and cytoplasmic FUS expression was elevated in CRPC (Fig. 8d and e). Our data therefore suggests that FUS is growth inhibitory in early stages of prostate cancer, in part via regulation of androgen signalling. However, since it is up-regulated in CRPC, it may shift to playing a different role in this stage of the disease.

**Fig. 9:**
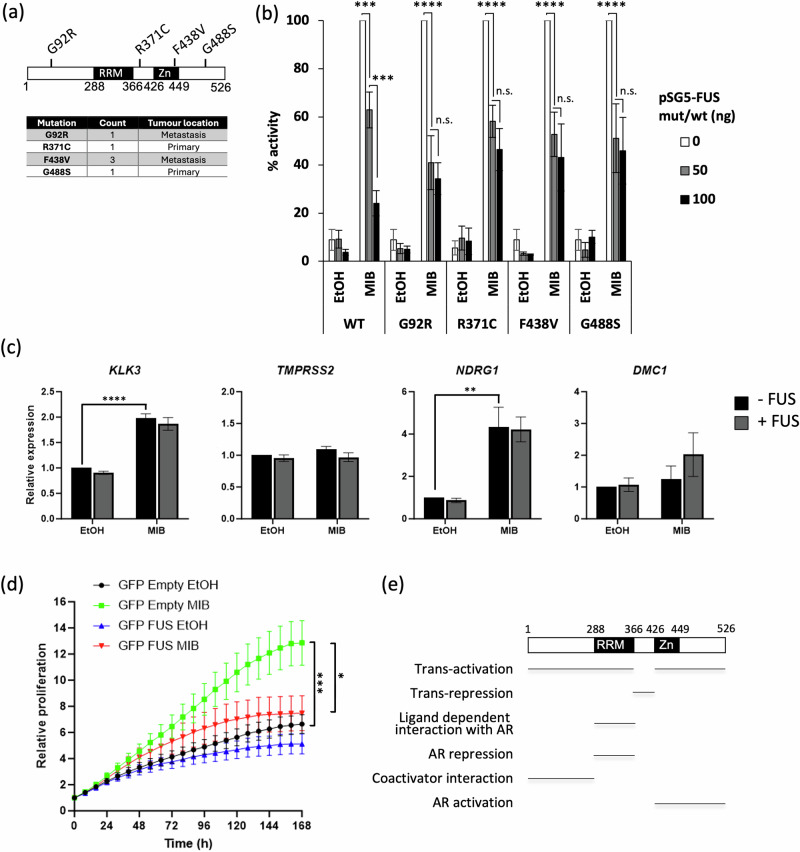
FUS regulation of AR gene expression appears to be lost in a model of CRPC (22RV1). **a** Schematic to show the position of the FUS mutations identified in PCa and summary of the mutation frequency and tumour location. **b** COS-1 cells were transfected with plasmids encoding the AR, wild-type or mutant FUS, a luciferase reporter (TAT-GRE-E1B-LUC) and β-galactosidase and treated ± MIB. Luciferase data was normalised to β-galactosidase activity. Mean of 3 independent repeats performed in duplicate ± 1SE. **c** 22RV1 were seeded in hormone-depleted media and transfected with pEGFP-empty or pEGFP-FUS plasmid and treated ± MIB. Target gene expression was quantified using qPCR. Mean of 3 independent repeats performed in duplicate ± 1SE. **d** 22RV1 cells were transfected/treated as for (**c**) and proliferation quantified using an Incucyte Cell Imaging System. Mean of 3 independent repeats performed in duplicate ± 1SE. **e** Schematic representation of FUS, highlighting the activation, repression and AR interaction domains identified. RRM = RNA Recognition Motif, Zn Zinc Finger. ANOVA ** *p* < 0.005, *** *p* < 0.0005, ****p < 0.0001. n.s. not significant.

To investigate this hypothesis further, the COSMIC database [49] was interrogated to identify FUS mutations in cancer. FUS mutations are rare in PCa (<0.3% of PCa samples in COSMIC), with 4 distinct somatic missense variants identified. Two of these (R371C, G488S) were detected in primary tumour samples, and 2 (G92R, F438V) in metastatic samples, with F438V observed in 3 individuals (Fig. 9a). As pathological FUS mutations in Amyotrophic Lateral Sclerosis (ALS) are associated with protein mislocalisation and cytoplasmic accumulation [50] we first checked cellular localisation of constructs carrying these alterations. The mutations linked to prostate cancer, did not affect FUS nuclear localisation, although some nuclear aggregates appeared to be present in some cells transfected with pEGFP-FUS_G488S_ (Supplemental Fig. 4). To investigate if these mutations alter the repressive effects of FUS upon AR, the effect of wild-type and mutant FUS expression upon AR activity was measured using reporter assays (Fig. 9b). In response to FUS-WT, AR activity was repressed in a dose dependent manner. All of the FUS mutants did significantly inhibit AR activity, however the higher concentrations did not further repress AR, i.e., it was not dose-dependent, and the maximal repression was lower than seen for the wild-type FUS. It therefore appears that FUS mutations found in PCa reduce the repressive activity of this protein.

To investigate the effect of FUS on AR activity in a more aggressive model of PCa, additional experiments were performed in 22RV1, a model of castrate-resistant disease which, in addition to the full-length AR, expresses the AR-V7 splice variant [51]. 22RV1 cells were transfected with pEGFP-C1-Empty or pEGFP-C1-FUS and expression and high transfection efficiency confirmed (Supplemental Fig. 5). qPCR was subsequently performed to analyse the effect of FUS upon the androgen-regulation of the target genes investigated previously. The expression of *KLK3* and *NDRG1* were significantly up-regulated in response to androgen, but in contrast to what was observed in LNCaP cells (Fig. 2a), FUS did not affect target gene expression (Fig. 9c). *TMPRSS2* and *DMC1* were not found to be significantly regulated by MIB and/or FUS. As FUS regulation of target genes appears to be lost in this line, we were interested to see if FUS has an effect upon the proliferation of 22RV1 cells. As expected, MIB enhanced the proliferation of 22RV1 cells and interestingly FUS still significantly inhibited proliferation (Fig. 9d). It therefore appears that in latter stages of PCa, FUS signalling pathways may be altered so that it has a reduced impact upon AR activity. However FUS still appears to retain tumour suppressive activity.

## Discussion

FUS is a multifunctional protein that has been linked to multiple diseases, including amyotrophic lateral sclerosis and cancer [15, 52]. We previously demonstrated that FUS has tumour suppressor activity in PCa [11], but contradictory to this, FUS had been shown to interact with and co-activate the AR [19]. To investigate this further, and to better understand by what mechanisms FUS inhibits PCa growth, the transcriptome of LNCaP PCa cells was analysed following up-regulation of FUS expression. This demonstrated that the overlap between FUS and AR target genes is significant and that, although FUS can act as a co-activator of the AR on some genes, overall it predominantly represses AR activity. Other co-factors have also been described to have differential effects upon transcription factor activity. For example, RIP140 has been shown to function both as a nuclear receptor coactivator and corepressor; the mechanisms of this dual activity remain to be fully elucidated but appear to be dependent upon cofactor interactions and promoter context [53]. It is therefore possible that FUS can act as both a co-activator and co-repressor. Analysis of H3K27Ac ChIP-Seq data suggest that FUS modulation of ARGs is associated with histone acetylation status. For example, the correlation of genes up-regulated by androgen with H3K27Ac was lost when FUS is present. Interestingly, genes that were found to only be androgen responsive in the presence of FUS were not associated with H3K27Ac. Investigation of additional histone modulations would be useful to further explore their relationship with FUS regulation of gene expression.

FUS has previously been shown to interact with the AR [19] and data presented here supports and extends this. Upon translocation to the nucleus, FUS and AR co-localise; the androgen-induced interaction is dependent upon the FUS RRM motif (Fig. 9e). Interestingly, this region was important in both androgen-dependent interaction and also in in the interaction between FUS and antiandrogen-bound AR. In reporter assays, FUS was found to repress androgen-activated AR and also enhance the antagonistic action of an antiandrogen. Importantly, FUS was found to interact with the AR LBD in the presence of the antiandrogen bicalutamide. This therefore suggests that FUS not only plays a role in the physiological (androgen) regulation of AR activity, but also influences pharmacological (anti-androgen) action and therefore may play a significant role in therapy response. This correlates with a study that used quantitative proteomics to identify cofactors that interact with agonist and antagonist bound oestrogen receptor-α [18]. In that study, FUS was found to interact with oestrogen receptor-α when it was bound to the anti-estrogen tamoxifen. Although the data presented here focus mainly upon the AR, we also found FUS to repress GR activity and to enhance ERα activity.

Co-repressors have been demonstrated to repress AR signalling via different mechanisms, for example via recruitment of histone deacetylases or inhibition of nuclear localisation [54]. Our detailed analysis of FUS domains provides insight into how this cofactor regulates AR activity. In agreement with a previous study [44], FUS was found to have an intrinsic N-terminal activation domain and we demonstrate this domain to interact with co-activators. In addition, we identified a novel C-terminal activation domain and a central trans-repression domain (Fig. 9e). The addition of trichostatin A, sodium butyrate and valproic acid demonstrates that this repression domain is not HDAC Class I- or II- dependent, although it is possible that other classes of deacetylases could be involved. However, the AR-repressive activity of FUS is not dependent upon this domain and instead the RRM motif, through which FUS interacts with the AR, is indispensable. This domain, and RNA binding, has also been shown to be important in the pathology of other diseases including Amyotrophic Lateral Sclerosis (ALS) [55].

Wang et al. demonstrated that FUS is recruited to the *CCND1* promoter via lncRNA and interacts with coactivators, including p300, to disrupt formation of the transcription initiation complex and therefore reduce gene expression [17]. Interestingly, in the absence of lncRNA, FUS has been found to adopt a compact structure, and RNA binding induces a conformational change that results in an extended structure that is thought to enable FUS to interact with CBP/p300, thereby facilitating transcriptional interference [56]. RNA binding and the RRM region are therefore of significant importance in FUS signalling. We demonstrated that the N-terminal activation domain of FUS can interact with other coactivators in addition to p300, namely SRC1 and ARA70. ChIP assays demonstrated that FUS inhibits receptor activity via an interesting mechanism. AR recruitment to the ARE within the regulatory region of *KLK3* is not affected by FUS, but SRC1 and RNA POLII recruitment is significantly impaired in response to this factor. It therefore appears that FUS can inhibition target gene expression by disrupting the AR transcriptional complex, rather than affecting AR binding itself.

Alterations in cofactor levels have been suggested as one mechanism of therapy resistance [57]. To investigate whether FUS levels are altered during disease progression, RNA-seq and quantitative proteomic analyses were performed in BPH, untreated primary PCa and CRPC patient samples. In agreement with our previous study [11], FUS protein levels are lower in PCa compared to BPH. This, combined with the findings of the current study, would suggest that FUS has an inhibitory role in early stages of PCa and is therefore downregulated to facilitate tumour growth. In contrast, FUS may have an oncogenic role in advanced, therapy resistant stages of the disease. The rationale behind this is that IHC analysis of patient samples showed FUS upregulated in CRPC, with an increase in cytoplasmic and nuclear expression. To investigate this, we analysed the effects of somatic missense FUS mutations that have been found in primary and metastatic PCa samples. Although these mutants were still able to repress AR activity, their repressive activity was muted compared to the wild-type protein. However, these mutations are rare in PCa (<0.3% of PCa samples in COSMIC) and so it remains unclear on how significant this is in advanced stages of the disease.

Analysis of FUS activity in a model of CRPC (22RV1) cells did provide interesting results. While in LNCaPs (a model of hormone-sensitive disease) we found that FUS reduced the AR induction of *KLK3* and enhanced regulation of NDRG1, in22RV1 cells, this regulation was completely lost, suggesting that FUS regulation of AR activity is altered/lost in advanced stages of the disease; although regulation of cellular proliferation was maintained. A likely explanation is that the altered expression and activity is a reflection of the plasticity associated with AR signalling throughout disease development and progression [58, 59]. For example, ChIP-seq analysis has demonstrated that the number of AR binding sites in the genome increases in prostate cancer compared to normal prostate and this is elevated further in CRPC [60]. Alterations in cofactor/pioneer factor expression, including FUS, are therefore likely to play an important role in reprogramming AR signalling, resulting in the gene signatures associated with the different stages of the disease [59].

In contrast to FUS, the other members of the TLS family (EWS and TAF15) were found to enhance AR activity in reporter assays, in agreement with previous studies showing that these factors have oncogenic activity in cancer [61, 62]. For example, Kedage et al. demonstrated that EWS interacts with oncogenic ETS factors to promote PCa progression [61] and EWS has been shown to act as a co-activator through interaction with CBP/p300 [63]. TAF15 has also been shown to have oncogenic properties in other tumours, such as gastric cancer [64]. Interestingly, similar to FUS, the expression of these factors was also found to be elevated in CRPC, suggesting that the entire TLS family may have an oncogenic role in driving advanced PCa. In addition to the TLS family, a number of other RNA binding proteins have been shown to be up-regulated during PCa progression [65]. Up-regulation of these functionally-related proteins appears to be as a result of increased ribosome biogenesis and chromatin-related functions that have been associated with CRPC.

In conclusion, FUS can modulate the activity of the AR in either direction, dependent on target, but appears to predominantly repress AR activity. The mechanism of repression is via disruption of the AR transcriptional complex and we propose a model by which tumour cells down-regulate this inhibitory factor in early stages of the disease. However, FUS levels are elevated in CRPC and it remains unclear as to the role of FUS in this stage of the disease. It appears that FUS regulation of AR activity is lost or altered in advanced stages of the disease and this could be a reflection of the plasticity associated with AR signalling throughout disease progression. Further work is needed to provide a complete understanding of the role that FUS plays in CRPC, including metastatic potential, and whether such alterations in activity can be targeted for therapeutic gain.

## Conclusion

FUS contains trans-activation and trans-repression domains and can act as both an androgen receptor coactivator and corepressor. FUS enhances the antagonistic activity of antiandrogens and predominantly acts as a corepressor of androgen receptor activity. The mechanism of repression appears to be disassembly of the transcriptional complex, blocking recruitment of coactivators and the basal transcription machinery. Analysis of patient samples demonstrated that FUS is down-regulated in early stages of prostate cancer and is up-regulated in advanced stages of the disease. Together, this suggests that FUS has an inhibitory role in early stages of the disease, at least in part as a result of repression of androgen receptor activity and is therefore down-regulated. However, in advanced stages of the disease, FUS regulation of AR activity appears to be lost, which may facilitate disease progression.

## Supplementary information

Supplementary Tables 1-4

Supplemental Figure 1. The effect of doxycycline upon gene expression in the parental LNCaP-TR2 line.

Supplemental Figure 2. Gene ontology analysis to identify pathways regulated by FUS and/or AR signalling.

Supplemental Figure 3. The AR co-localises with FUS in the nucleus.

Supplemental Figure 4. Mutations of FUS, associated with prostate cancer, do not affect protein cellular localisation.

Supplemental Figure 5. Confirmation of FUS over-expression and transfection efficiency in 22RV1 cells.

## Data Availability

The datasets used and/or analysed during the current study are available from the corresponding author on reasonable request.
