# Supplementary material for: Disruption of androgen receptor-cofactor interactions by the RNA-binding protein FUS/TLS alters androgen signalling in prostate cancer
Source: Oncogene. 2026 Feb 6;45(8):757–73. doi: 10.1038/s41388-026-03682-3 (PMC12909129; doi:10.1038/s41388-026-03682-3)
Supplement: Supplementary file 1 — Supplementary Tables 1-4 [file 41388_2026_3682_MOESM1_ESM.docx]

**Supplementary Table 1. Cloning primer sequences used for the insertion of FUS full-length and truncations into the pM-GAL4 plasmid**

|  | Primer (5’-3’) | |
| --- | --- | --- |
| FUS construct | Forward (*Bam H*I) | Reverse (*Xba* I) |
| Full-length | CGGGATCCCGATGGCCTCAAACGA | GCTCTAGAGCTAATACGGCCTCTCC |
| A | CGGGATCCCGATGGCCTCAAACGA | GCTCTAGAGCGACCTTGATAGGATT |
| B | CGGGATCCCGATGGCCTCAAACGA | GCTCTAGAGCGGCCTTACACTGGTT |
| C | CGGGATCCCGTTTGTGCAAGGCCTG | GCTCTAGAGCTAATACGGCCTCTCC |
| D | CGGGATCCCGTGGAAGTGTCCTAAT | GCTCTAGAGCTAATACGGCCTCTCC |
| E | CGGGATCCCGCTATTTGCTACTCGC | GCTCTAGAGCTAATACGGCCTCTCC |

**Supplementary Table 2. Cloning primer sequences used for the insertion of TAF15 into the pSG5 plasmid**

|  | Primer (5’-3’) | |
| --- | --- | --- |
|  | Forward (*Bam H*I) | Reverse (*Xma* I) |
| TAF15 | TCCCCCCGGGGGGAACCATGTCGGATTCT | CGGGATCCCGTCAGTATGGTCG |

**Supplementary Table 3. Primers used for site directed mutagenesis of the pSG5-FUS and pEGFP-C1-FUS plasmids**

|  | Primer (5’-3’) | |
| --- | --- | --- |
| FUS mutation | Forward | Reverse |
| G92A | GGAGGACTGCTGCCTGTAAGACGATTGGG | CCCAATCGTCTTACAGGCAGCAGTCCTCC |
| F438V | CATTCATTCCTCCAAGAGACGTTCATATTCTCACAGGTG | CACCTGTGAGAATATGAACGTCTCTTGGAGGAATGAATG |
| G488S | CGGTCCCCGCTGCGGCCCCGG | CCGGGGCCGCAGCGGGGACCG |
| K510E | CCTGGAATCCATCTCGCCAGGGCCAAAGC | GCTTTGGCCCTGGCGAGATGGATTCCAGG |

**Supplementary Table 4. qPCR primer sequences.**

|  | Primer (5’-3’) | |
| --- | --- | --- |
|  | Forward | Reverse |
| *DMC1* | CTTTCCGTCCAGATCGCCTTA | CGTGCATAAAGTACGTTGTCCA |
| *NDRG1* | GCAGCACACACTTCACAAAGC | CCAGGCACCCGTTTGAAC |
| *FUS* | GCAGGGAGAGGCCGTATTA | CTTGGGTGATCAGGAATTGG |
| *L19* | GCAGCCGGCGCAAA | GCGGAAGGGTACAGCCAAT |
| *KLK3* | TTGTCTTCCTCACCCTGTCC | AGCTGTGGCTGACCTGAAAT |
| *TMPRSS2* | AATCGGTGTGTTCGCCTCTAC | GCGGCTGTCACGATCC |
