# Supplementary figures and images for: Disruption of androgen receptor-cofactor interactions by the RNA-binding protein FUS/TLS alters androgen signalling in prostate cancer
Source: Oncogene. 2026 Feb 6;45(8):757–73. doi: 10.1038/s41388-026-03682-3 (PMC12909129; doi:10.1038/s41388-026-03682-3)

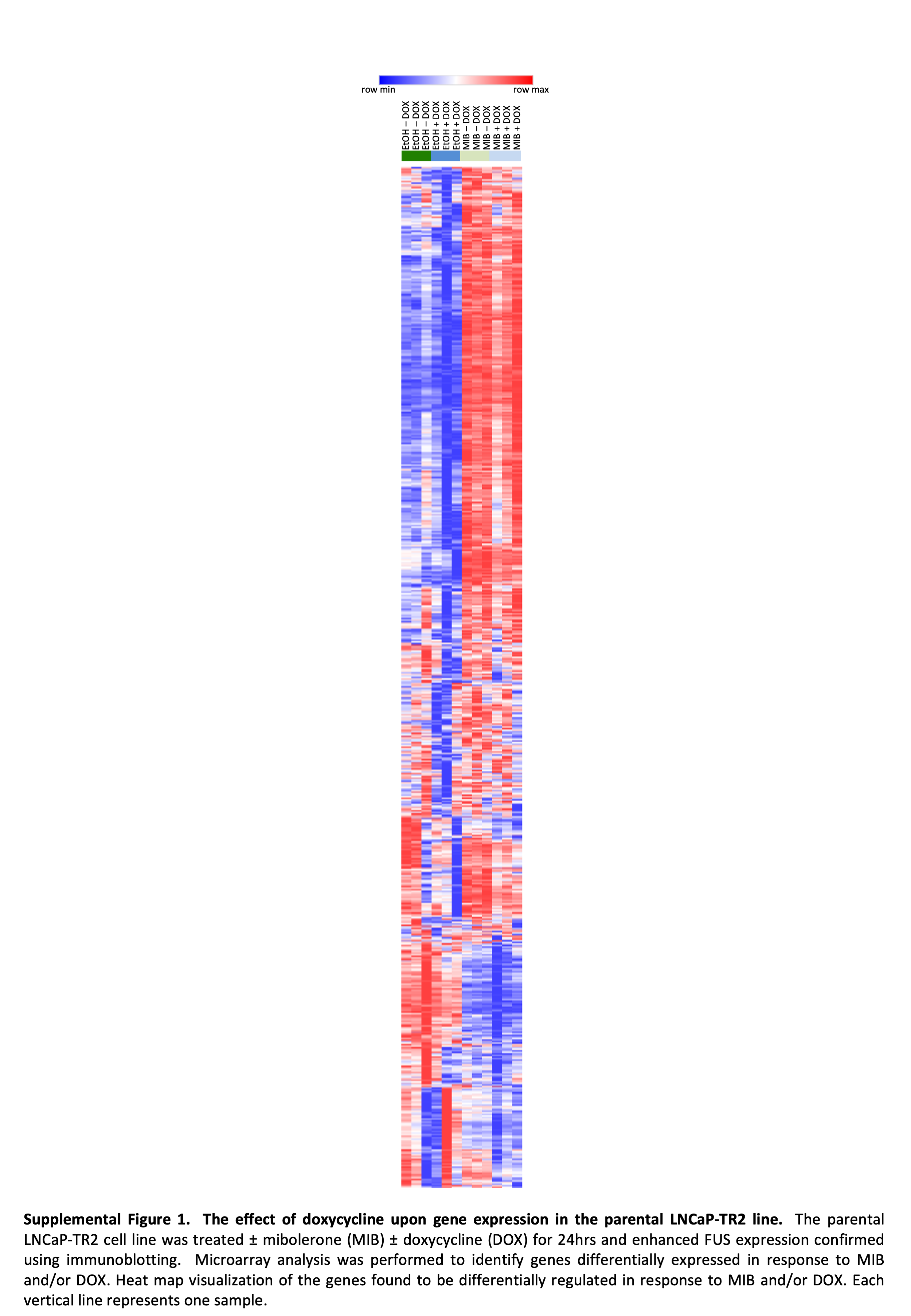

Supplement: Supplementary file 2 — Supplemental Figure 1. The effect of doxycycline upon gene expression in the parental LNCaP-TR2 line. [file 41388_2026_3682_MOESM2_ESM.tif]

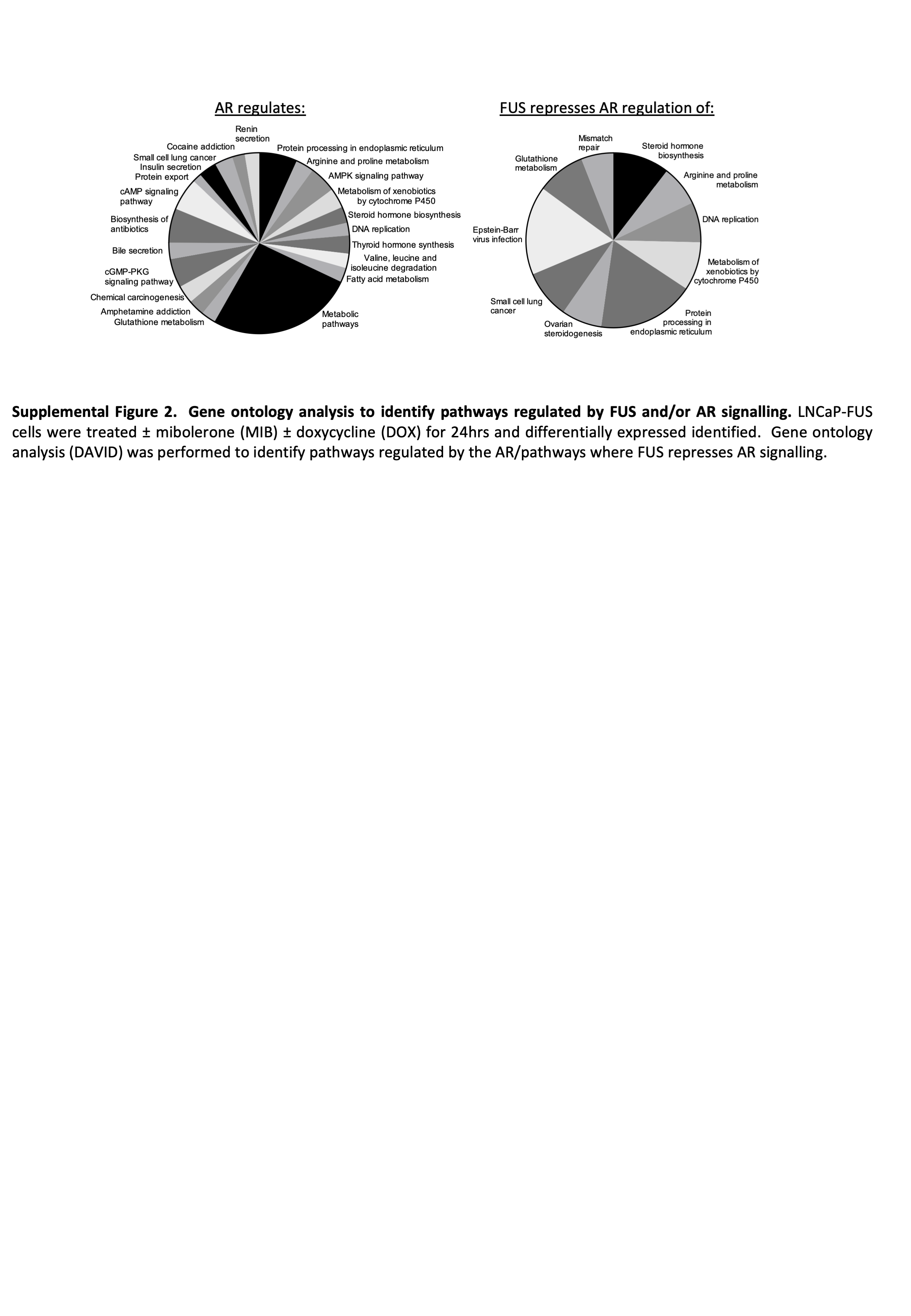

Supplement: Supplementary file 3 — Supplemental Figure 2. Gene ontology analysis to identify pathways regulated by FUS and/or AR signalling. [file 41388_2026_3682_MOESM3_ESM.tif]

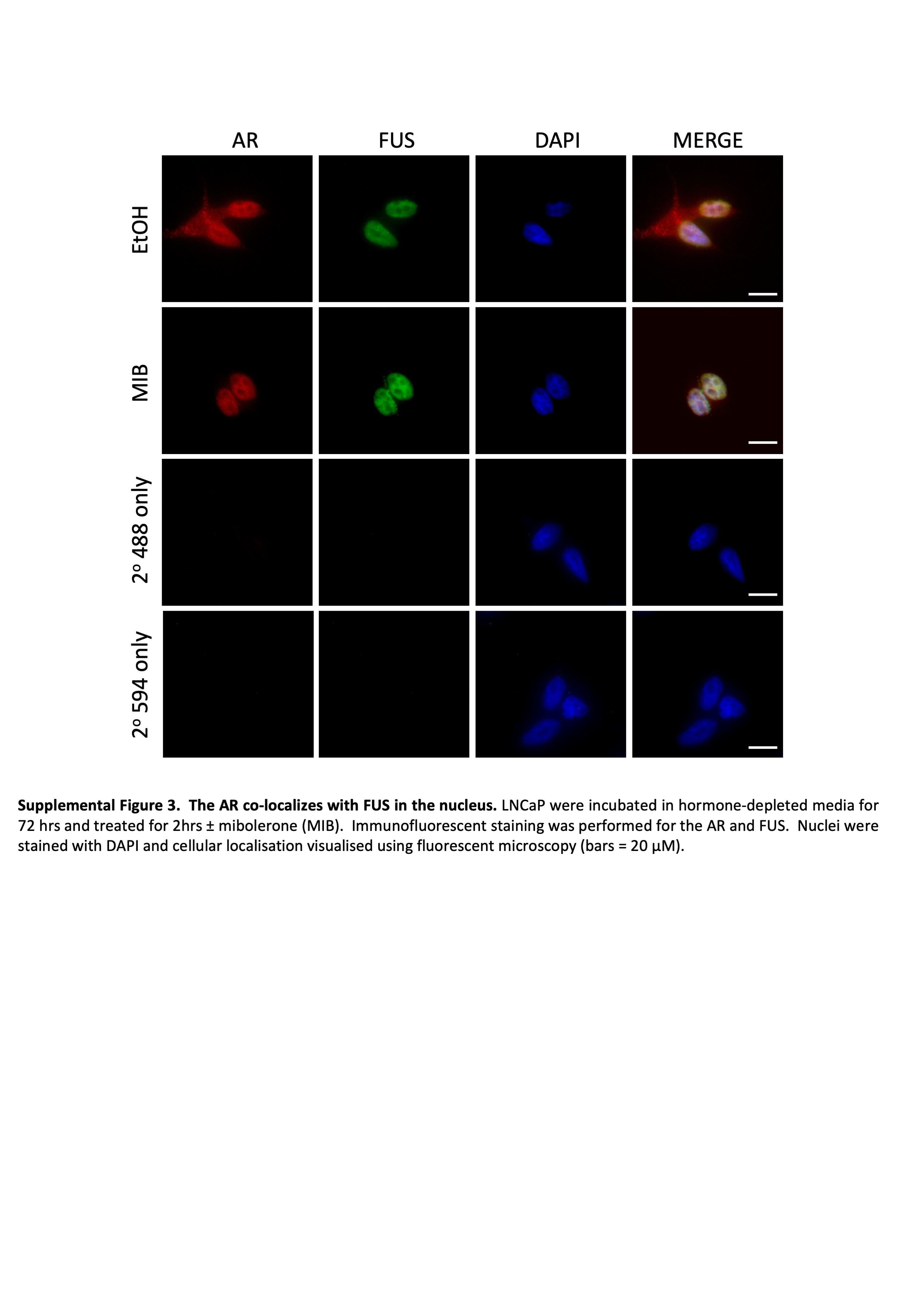

Supplement: Supplementary file 4 — Supplemental Figure 3. The AR co-localises with FUS in the nucleus. [file 41388_2026_3682_MOESM4_ESM.tif]

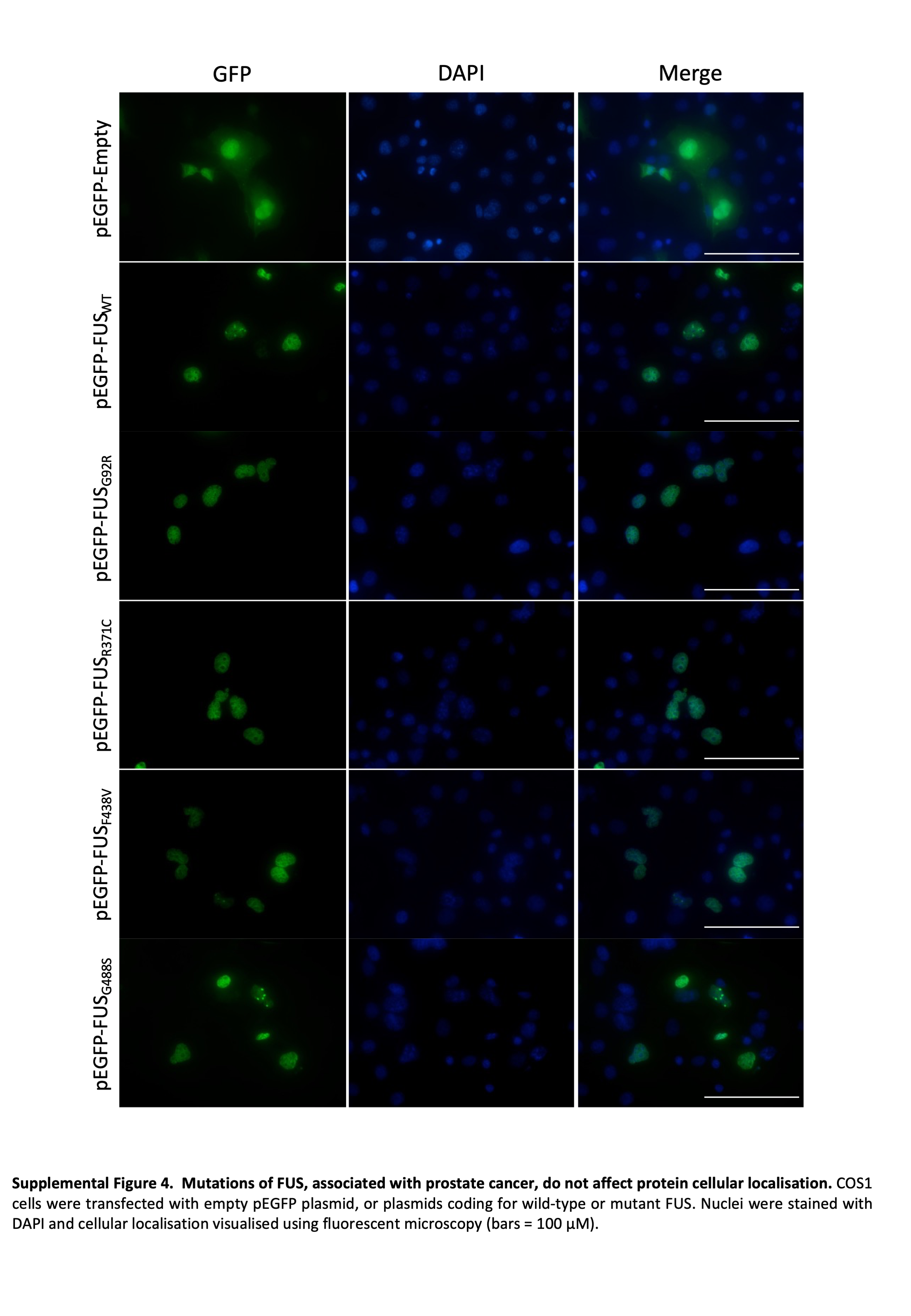

Supplement: Supplementary file 5 — Supplemental Figure 4. Mutations of FUS, associated with prostate cancer, do not affect protein cellular localisation. [file 41388_2026_3682_MOESM5_ESM.tif]

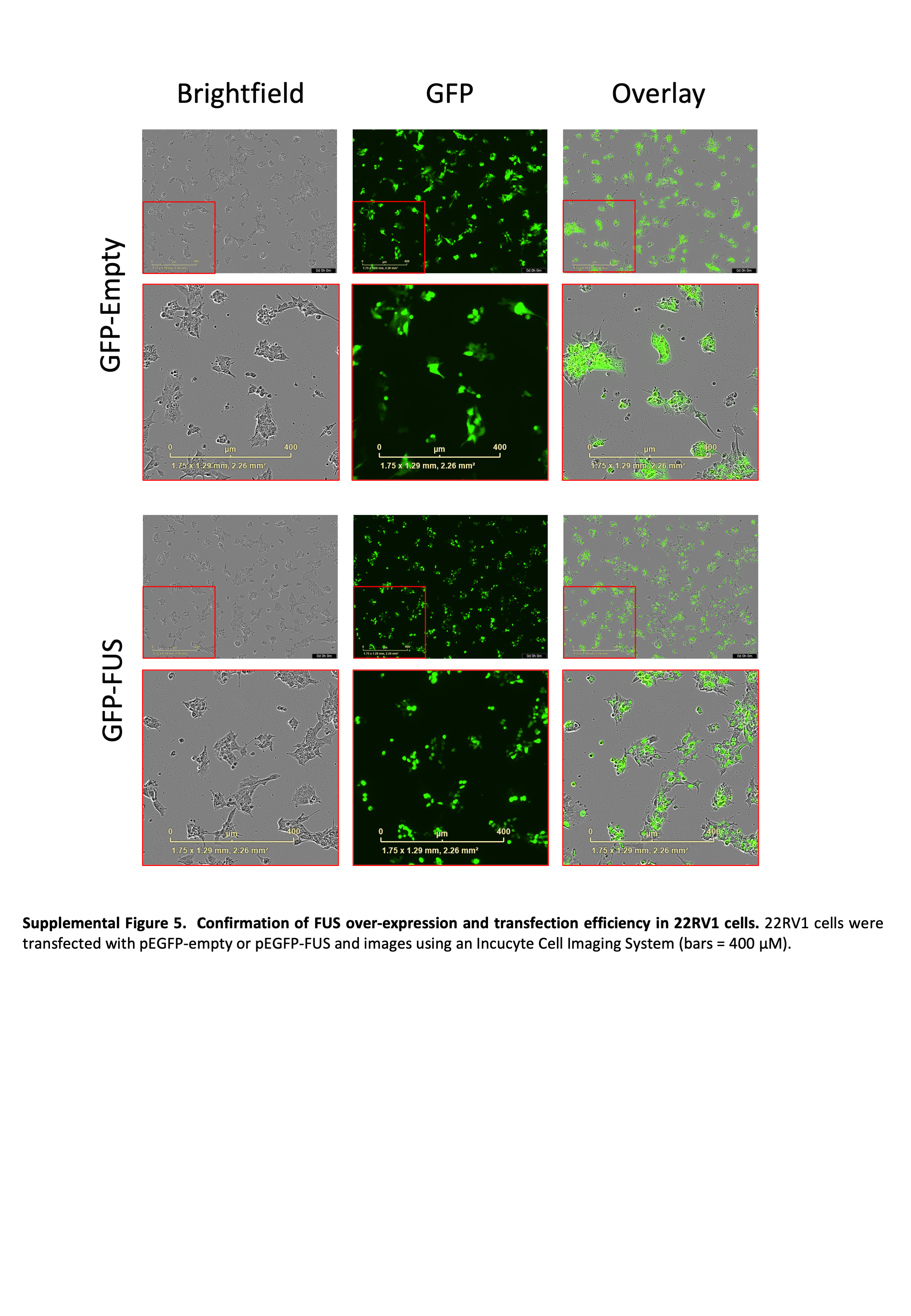

Supplement: Supplementary file 6 — Supplemental Figure 5. Confirmation of FUS over-expression and transfection efficiency in 22RV1 cells. [file 41388_2026_3682_MOESM6_ESM.tif]
